# Panton-Valentine Leukocidin Genes in *Staphylococcus aureus*

**DOI:** 10.3201/eid1207.050865

**Published:** 2006-07

**Authors:** Damian C. Melles, Willem B. van Leeuwen, Hélène A.M. Boelens, Justine K. Peeters, Henri A. Verbrugh, Alex van Belkum

**Affiliations:** * University Medical Center Rotterdam, Rotterdam, the Netherlands

**Keywords:** Panton-Valentine Leukocidin, Staphylococcus aureus, letter

The pathogenicity of *Staphylococcus aureus* depends on various bacterial surface components and extracellular proteins. However, the precise role of single virulence determinants in relation to infection is hard to establish. The frequent recovery of staphylococcal isolates that produce leukocidal toxins from patients with deep skin and soft tissue infections, particularly furunculosis, cutaneous abscesses, and severe necrotizing pneumonia, suggests that the Panton-Valentine leukocidin (PVL) is 1 such virulence factor that has a major role in pathogenicity (*1**–**3*).

In 1932, Panton and Valentine described PVL as a virulence factor belonging to the family of synergohymenotropic toxins (*4*). These toxins form pores in the membrane of host defense cells by synergistic action of 2 secretory proteins, designated LukS-PV and LukF-PV, which are encoded by 2 cotranscribed genes of a prophage integrated in the *S. aureus* chromosome (*5*). PVL is mostly associated with community-acquired methicillin-resistant *S. aureus* (MRSA) infections and distinguishable from nosocomial MRSA by nonmultidrug resistance and carriage of the type IV staphylococcal chromosome cassette element (SCC*mec* type IV) (*6**,**7*).

Despite the presumed importance of PVL as a virulence factor, few data are available on its prevalence among *S. aureus* isolates from the nares of healthy persons compared with stains isolated from infections. This lack of data led us to investigate the frequency of PVL gene–positive *S. aureus* strains obtained from the nares of healthy carriers in the community. For this purpose, a single polymerase chain reaction method was used to detect both *lukS-PV* and *lukF-PV* genes (*2*).

In a previous study, the population structure of *S. aureus*, isolated from the nares of healthy persons in the Rotterdam area, the Netherlands, was elucidated (*8*). Strains were obtained from healthy children (<19 years) and elderly persons (>55 years). Invasive strains (blood culture, skin and soft tissue infections, and impetigo isolates) were included in this study (Table). All carriage and clinical isolates (n = 1,033) were *mecA* negative. We used the same strain collection to study the PVL prevalence in carriage and invasive isolates of *S. aureus* from a single geographic region.

**Table:** Panton-Valentine Leukocidin (PVL) distribution among carriage and invasive isolates per genetic cluster of *Staphylococcus aureus*

	Amplified fragment length polymorphism cluster					
	I (n = 462)	II (n= 261)	III (n = 208)	IVa (n = 62)	IVb (n = 40)	Total (N = 1,033)
	PVL positive, n (%)					
Carriage isolates (n = 829)	1	1	1	0	2	5 (0.6)*
Bacteremia isolates (n = 146)	1	1	0	0	1	3 (2.1)†
Soft tissue infection isolates (n = 18)	1	5	0	1	0	7 (38.9)‡
Impetigo isolates (n = 40)	0	0	0	0	0	0 (0.0)
Total (N = 1,033)	3 (0.6)§	7 (2.7)	1 (0.5)¶	1 (1.6)	3 (7.5)#	15 (1.5)
MLST** data of PVL-positive isolates	CC 1, n = 2 CC 8, n = 1	CC 30, n = 7	CC 45, n = 1	CC 22, n = 1	CC 121, n = 3	

‡versus *Fisher exact test (2-sided); p<0.0001. ‡versus †Fisher exact test (2-sided); p<0.0001. #versus §Fisher exact test (2-sided); p = 0.0079. #versus ¶Fisher exact test (2-sided); p = 0.0140. **MLST, multilocus sequence typing; CC, clonal complex.

Five PVL-positive *S. aureus* strains (0.6%) were found in the carriage group (n = 829), and 3 (2.1%) of 146 blood-culture isolates carried the PVL gene (Table). This finding is in agreement with previously reported low PVL prevalences by Prevost et al. (0% in 31 carriage isolates and 1.4% in 69 blood-culture isolates) and Von Eiff et al. (1.4% in 210 carriage isolates and 0.9% in 219 blood-culture isolates) (*9**,**10*). However, a higher prevalence of PVL (38.9%) was found in *S. aureus* strains causing abscesses and arthritis (Fisher exact test, p <0.0001) (*8*). This finding is also in agreement with the proposed involvement of PVL in severe and invasive (soft tissue) staphylococcal infections (*1**–**3*). No significant differences were found in the presence of PVL when carriage isolates were compared with invasive blood-culture isolates. PVL was found in each major genomic amplified fragment length polymorphism (AFLP) cluster, indicating that PVL has been introduced in distinct phylogenetic subpopulations of *S. aureus* (Figure). Multilocus sequence typing analysis of a subset of the strain collection showed that the 15 PVL-positive strains were within clonal complex (CC) 30 (n = 7), CC 121 (n = 3), CC 1 (n = 2), CC 8 (n = 1), CC 22 (n = 1), and CC 45 (n = 1) (Table) (*8*). Although PVL was found among several staphylococcal genotypes, it was slightly overrepresented in AFLP cluster IVb (CC 121) compared with major clusters I and III. Whether the prevalence of PVL in carriage- and blood-culture isolates is higher and differs among distinct genetic clusters of *S. aureus* in countries with endemic CA-MRSA has to be investigated further.

**Figure:**
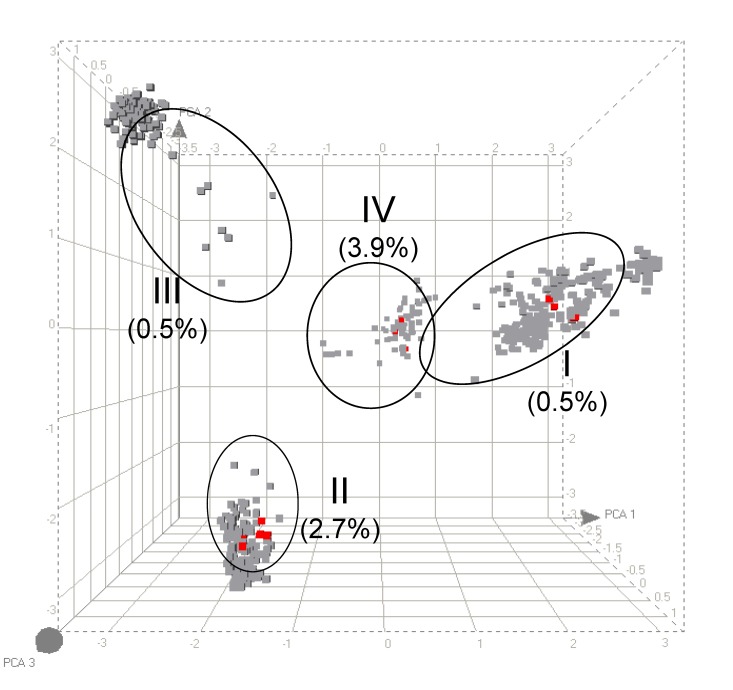
Principal component analysis of amplified fragment length polymorphism (AFLP) data of 1,033 *Staphylococcus aureus* strains. The different cubes (plotted in 3-dimensional space) represent every strain in the study. Each axis represents the score calculated for that strain on each principle component. The 4 circles indicate the different genetic AFLP clusters. Cluster IV could be subdivided in 2 minor clusters (*8*). The percentages (below each cluster) indicate the relative number of Panton-Valentine leukocidin (PVL)-positive strains in each major AFLP cluster. The single PVL-positive strain in cluster III is not visible behind the gray cubes. Gray, PVL-gene negative; red, PVL-gene positive.

In conclusion, we have shown that the PVL-encoding phage has entered distinct staphylococcal lineages, although its prevalence differs per clonal group. PVL is associated with skin and soft tissue infections but not with bacteremia, which suggests that PVL is not likely to be involved in the pathogenesis of bacteremia. Infections caused by PVL-positive *S. aureus* strains have been documented since the 1930s. Expansion and increased incidence of such infections, however, are more recent, and further epidemiologic studies for tracking this phenomenon are still warranted.
